# Femoral Neck Version Affects Medial Femorotibial Loading

**DOI:** 10.1155/2013/328246

**Published:** 2013-02-21

**Authors:** T. A. Papaioannou, Georgios Digas, Ch. Bikos, V. Karamoulas, E. A. Magnissalis

**Affiliations:** ^1^Department of Orthopaedics, General Hospital of Xanthi, 67100 Xanthi, Greece; ^2^BioHexagon Ltd., Varnis 36, 17124 Athens, Greece

## Abstract

The aim of this study was to provide a preliminary evaluation of the possible effect that femoral version may have on the bearing equilibrium conditions developed on the medial tibiofemoral compartment. A digital 3D solid model of the left physiological adult femur was used to create morphological variations of different neck-shaft angles (varus 115, normal 125, and valgus 135 degrees) and version angles (−10, 0, and +10 degrees). By means of finite element modeling and analysis techniques (FEM-FEA), a virtual experiment was executed with the femoral models aligned in a neutral upright position, distally supported on a fully congruent tibial tray and proximally loaded with a vertical only hip joint load of 2800 N. Equivalent stresses and their distribution on the medial compartment were computed and comparatively evaluated. Within our context, the neck-shaft angle proved to be of rather indifferent influence. Reduction of femoral version, however, appeared as the most influencing parameter regarding the tendency of the medial compartment to establish its bearing equilibrium towards posteromedial directions, as a consequence of the corresponding anteroposterior changes of the hip centre over the horizontal tibiofemoral plane. We found a correlation between femoral anteversion and medial tibiofemoral compartment contact pressure. Our findings will be further elucidated by more sophisticated FEM-FEA and by clinical studies that are currently planned.

## 1. Introduction

Osteoarthritis at large and especially in the knee joint is believed to be the result of local factors acting within the context of a systemic susceptibility [1, 2].

These local factors govern how load is distributed across the articular cartilage. It is the distribution of load that confers upon weight bearing joints the ability to bear loads that are several times greater than body weight over a lifetime [3]. Because alteration in these local factors may lead to the development of excessive stresses on the joint and cause damage to the articular cartilage, they are receiving increasing attention in studies of the natural history of OA and especially the malalignment and laxity.

The connection between varus and valgus deformities and gonarthrosis is well known [4]. Very few reports exist concerning the relation between the torsional element of the femur (anteversion) and the development of knee osteoarthritis. In a cadaveric study [5], the correlation between increasing arthritis of the knee and decreasing femoral anteversion has been identified. In a clinical study [6], the existence of femoral torsional malalignment syndrome in arthritic knees with involvement of the patellofemoral joint has been confirmed. In a recent cadaveric study by Bretin et al. [7], the tibiofemoral joint centre of force measured by intra-articular pressure sensor moved medially with external femoral malrotation caused by medial displacement of the mechanical axis. This rotational malalignment would result in abnormal loading of the articular cartilage of the knee [8].

Several studies showed that the results of tibia osteotomy used to treat osteoarthritis of the medial compartment of the knee deteriorate over time even when the initial correction is optimal [9, 10]. The extent of femoral anteversion is one factor that correlates with long-term success of a valgus tibia osteotomy [11].

The proximal metaphysis and neck are anteverted in relationship to the posterior aspect of the femoral condyles by approximately 15°. The current study was designed to investigate the correlation between femoral anteversion and bearing conditions on the medial compartment of the knee. It was decided to use computational mechanics and execute a preliminary virtual experiment to study the possible effect that femoral version may have on the bearing equilibrium conditions of the medial tibiofemoral contact. We used finite element modeling and analysis techniques (FEM-FEA) in biomechanical scenarios involving vertically loaded femoral models of varying neck orientations, under upright static limb alignment.

## 2. Materials and Methods

### 2.1. Rationale, Design, and Assumptions of the Virtual Experiment

The main objective of the reported study was to provide a preliminary assessment of the prevailing bearing equilibrium conditions on the medial compartment of the knee, as resulting from different anteversion angles; as no previous such assessment was found reported, it was decided to execute this virtual experiment in a rather conservative manner, involving several assumptions as follows.

- Identical digital 3D femoral models would be adopted, only differing in their anteversion and additionally neck-shaft angles, in order to “freeze” other morphological parameters.
- All femoral models would be aligned in space, according to a recognized neutral upright configuration, in order to allow proximal morphological variations to uniformly demonstrate their relative positioning over the horizontal tibiofemoral compartments. The posterior aspects of the femoral condyles and the base of the greater trochanter rest on a vertical plane, while the femoral condyles rest on a horizontal plane [12].
- All femoral models would be proximally loaded with a simplified vertical-only hip load, in order to prevent cross-effects from AP and ML transverse load components. The femoral head was loaded with a vertical force of 2800 N, equivalent to about three times body weight [13] applied through the hip joint center, which was individually established for each model with an accuracy of ±1 mm.
- All femoral models would be distally supported on the same fixed tibial component model, providing identical horizontal and fully congruent tibiofemoral compartments, in order to ensure constant contact areas and thus justify comparisons between tendencies in changing tibiofemoral bearing equilibrium conditions. After a series of pilot virtual experiments, a depth of 4 mm in both compartments was found adequate for highlight bearing conditions. Under these conditions, it was found that all loaded models would remain in equilibrium and simultaneously in sufficient congruity and load bearing contact against the tibial tray.

### 2.2. Femoral Model and Morphological Variations

A digital 3D solid model of the left physiological adult femur was used for the purposes of this study. In order to create individual morphological variations, this model was manipulated using state-of-the-art 3D CAD software tools (SolidWorks version 2007). Thus, out of this initial femoral model, seven [11] otherwise identical morphological variants were generated only differing by combinations of different neck-shaft angles (varus 115, normal 125, and valgus 135 degrees) as well as version angles (+10, 0, and −10 degrees) as controlled and measured on frontal and transverse planes, respectively. Hence, for this virtual experiment, three (3) groups of three (3) models were compiled (Table 1).

### 2.3. Material Properties

Materials were considered isotropic, and the mechanical properties were assigned, following the formulation set by Peng et al. [14]. Femoral models were assumed as cortical shells, with an elastic modulus of 17.6 GPa and a Poisson's ratio of 0.30. The material of the tibial tray was arbitrarily chosen as polyethylene with an elastic modulus of 1.0 GPa and a Poisson's ratio of 0.42.

### 2.4. Finite Element Modeling and Analysis (FEM-FEA)

**Figure 1:**
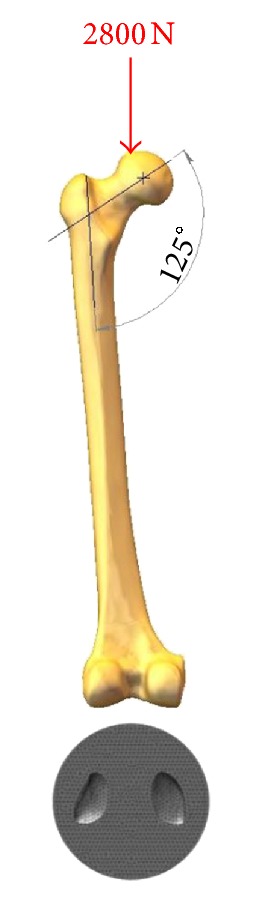
The femoral models were aligned in neutral upright configuration and were loaded with a simplified vertical force of 2800 N. All models were distally supported on the same fixed tibial component model.

In this virtual experiment, FEM-FEA was conducted using pertinent software tools (CosmosWorks version 2007) with static linear analysis. All models were meshed using approximately 180,000 tetrahedral elements of a size ranging down to 0.9 mm at the region of the femoral condylar contacts, where higher resolution was sought. The virtual experiment with a representative loaded model is shown in Figure 1.

### 2.5. Quantitative Results

For the comparisons meant to be addressed by this virtual experiment, three quantitative results were to be assessed: (a) the coordinates of the hip centre trace on the horizontal tibiofemoral plane, reflecting alterations in the leverarms of the hip joint load, (b) the computed equivalent von Mises stresses on the medial tibiofemoral condyle, reflecting tendencies in changing of the bearing equilibrium conditions, under the adopted neutral upright alignment, and (c) the percentage of condylar areas covered by different levels of stress, when projected on plan views of the medial tibiofemoral condyle.

For this latter parameter, based on pilot virtual experiments, two technical decisions were made in order to facilitate the above described and justified comparative assessment: a stress scale of 0 to 5 MPa was adopted as the one most revealing differences between colored stress distributions of the models; within this scale, stress values were categorized as “low” (<1.25 MPa, shown blue), “medium” (1.25 to 3.75 MPa, shown green), and “higher” (>3.75 MPa, shown red).

## 3. Results

**Figure 2:**
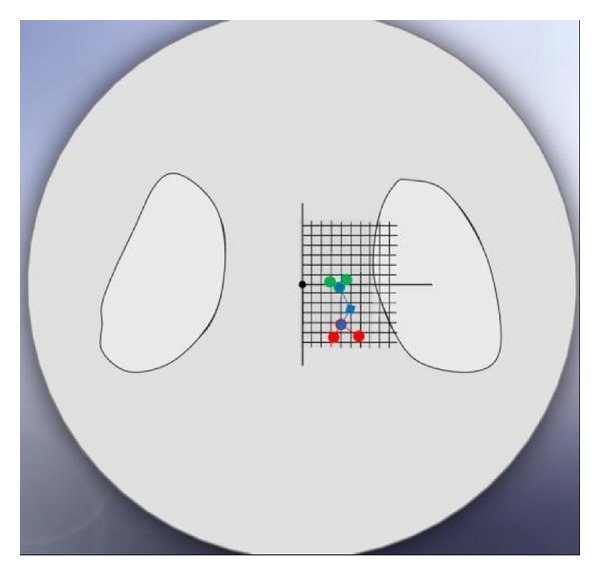
The coordinates of the hip centre trace when projected on plan views of the medial tibiofemoral condyle, reflecting alterations in the lever arms of the hip joint load.

For all femoral models, Figure 2 shows the coordinates of the hip centre trace on the horizontal tibiofemoral plane, reflecting alterations in the leverarms of the hip joint load. It can be appreciated that larger travels of the hip centre trace are associated with changes of the femoral version angle (blue line travel of up to 8 mm in the anteroposterior direction or sagittal plane, group 1), while for changes in neck-shaft angle, the travels of the hip centre trace are lower (in the mediolateral direction or coronal plane), both for retroversion angle (red line, group 2) and normal version angle (green line, group 3). In the interpretation of these findings, one must bear in mind that hip joint centres were numerically established for each model with an accuracy of ±1 mm.

**Figure 3:**
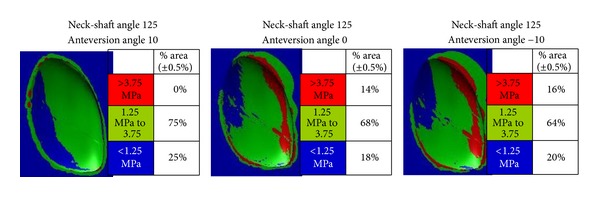
The percentage of condylar areas covered by different levels of stress and their topographical distributions when projected on plan views of the medial tibial tray for group 1 with normal neck-shaft angle and variable anteversion angle.

**Figure 4:**
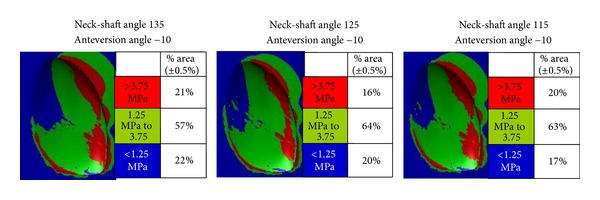
The percentage of condylar areas covered by different levels of stress and their topographical distributions when projected on plan views of the medial tibial tray for group 2 with retroversion angle of 10° and variable neck-shaft angle.

**Figure 5:**
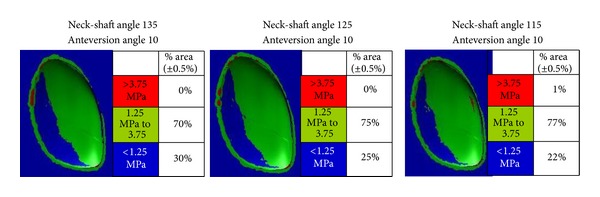
The percentage of condylar areas covered by different levels of stress and their topographical distributions when projected on plan views of the medial tibial tray for group 3 with anteversion angle of 10° and variable neck-shaft angle.

As clearly shown in Figure 3, it can be appreciated that reduction of the femoral version angle from normal to zero and then retroversion is associated with a substantial increase of area with high stresses at the medial and posterior quadrants of the compartment under study (0% at 10° anteversion, 14% at neutral position, and 16% at 10° retroversion, Table 2). In addition to that, femoral retroversion proves such a prevailing parameter that the above-mentioned posteromedially accentuated trends are not moderated by changes in the neck-shaft angle (Figure 4). When the retroversion angle was kept constant, the lower percentage (16%) of the area where higher stresses were developed was recorded with the normal neck-shaft angle (125°). Valgus and varus configurations increased the higher stress area to 21% and 20%, respectively (Figure 4, Table 2). Indeed, as shown in Figure 5, neck-shaft angle has a rather small effect and proves to be of minor importance for the femoral models with 10° of anteversion (Table 2).

## 4. Discussion

In knee osteoarthritis (OA), the medial tibiofemoral compartment is the most common site of disease. During activities of daily living, the medial side of the knee is loaded about 50% more than the lateral side of the knee. This relative difference is due to the adduction moment normally produced at the knee during weight bearing [15].

Conditions that increase the stress on the articular surface of the knee can lead to mechanical and biological breakdown of the articular cartilage [16].

The interrelation between biomechanical abnormalities and knee OA is complex. Conditions that increase the stresses across a smaller surface area lead to a circle of progressive articular cartilage degeneration and OA [17].

The knee joint does not function in isolation from the rest of the lower limb kinematic chain during weight-bearing activities. Hip and ankle/foot mechanics may influence knee joint during gait. The lower-limb torsion contributes together with coronal malalignment to the development of single-compartment knee osteoarthritis. While the relationship between medial tibiofemoral OA and external tibial torsion has been recognized [18–21], there is no conclusion about the relationship between femoral neck rotation and medial knee OA [22].

Some authors hypothesize on a correlation between femoral torsion error and axis deviation or torsion error and arthrosis of the knee joint [5, 23, 24].

A recent cadaveric study by Kenawey et al. [19] showed that decreased femoral neck anteversion was associated with increased pressure of the medial knee compartment up to 28,5% at 20° of retroversion compared to the pressure at 20° anteversion. These results are in agreement with our findings. Under normal neck-shaft angle (125°), the amount of anteversion is the main factor governing the percentage of medial condylar area prone to higher stresses. A normal anteversion of 10° eliminates this area, while no anteversion at all or retroversion (−10°) increases it (to 14% and 16%, resp.). Both studies show that decreasing the femoral neck version increases the stresses on the medial tibia compartment.

We found that neck-shaft angle proved to be of rather indifferent influence with respect to bearing equilibrium conditions in the medial knee compartment. Reduction of femoral version, however, appears as the most influencing parameter regarding the tendency of the medial compartment to establish its bearing towards posteromedial directions, as a consequence of the corresponding anteroposterior changes of the hip centre over the horizontal tibiofemoral plane. Obviously, in this virtual experiment, no ligamentous structures or femoral bowing was adopted that could resist such tendencies. However, based on these preliminary findings, it is believed that under reduced femoral version or retroversion, even in dynamic gait conditions, such tendencies although resisted would indeed exist, mainly, as a consequence of prevailing differences in the mechanical axis alignment which is expected to lead to the establishment of a more posteromedial initial equilibrium. This hypothesis will nonetheless be addressed with more sophisticated future computational and clinical studies.

Our results support recent findings [11], where the long-term outcomes of tibia valgus osteotomy for medial compartment knee OA were evaluated. Femoral retroversion was significantly greater in patients in whom valgus decreased over time than in those in whom valgus increased over time.

Our findings suggest that there is more tolerance in the knee to internal rotation (intoeing) due to anteversion than to external rotation (outtoeing) caused by retroversion. The significance of this relationship between femoral anteversion and higher stresses developed on the medial compartment of the knee may have an application to the treatment of rotational deformity in children. Children with increased anteversion are unlikely to develop arthritis with maturity. On the other hand, those children maturing to adulthood with less than normal anteversion may be at risk for the future development of medial OA of the knee.

The limitations of our study must be recognized, with respect to the biomechanical breadth and fidelity of our simulation. We believed that the adopted approach, despite limitations (in strict simulation or biomechanical terms), allows for a sound preliminary comparative assessment. Methodological improvements could include more realistic anatomical configurations (e.g., tibia torsion and femoral bowing) and loading conditions (3D hip load, ligaments, and muscles). On the other hand, except femoral neck version, several others parameters as weight, gender, activities, genetic factors, and other mechanical factors may also affect the different stress distributions on the medial compartment of the knee leading to OA in some patients. The correlation between these parameters is still unknown.

However, within the exact context of our study, the relative sense and comparative significance of our preliminary findings suggested that in cases of retroversion and regardless of the neck-shaft angle, the area prone to higher stresses is expected to be established more posteromedially, compared to anteversion. If future studies confirm our findings, surgical changes in hip anatomy, such as osteotomy, change of anteversion in hip replacement, or even femoral fracture healed in rotation might influence biomechanics so that progression and even development of knee osteoarthritis will become more or less likely to happen.

Our findings raise the hypothesis that the development of medial knee OA is facilitated by certain anatomical abnormalities in the hip region. Further computational and clinical studies based on computerized tomography and gait analysis, where patients with different femoral neck version are correlated to the existence of medial knee OA, are necessary to elucidate this issue.

## Figures and Tables

**Table 1:** The neck-shaft and anteversion angles of models in the three groups to be studied.

Group 1: three models with neutral neck-shaft angle of 125°	125° +10°	125° 0°	125° −10°
Group 2: three models with retroversion angle of −10°	135° −10°	125° −10°	115° −10°
Group 3: three models with neutral anteversion angle of +10°	135° +10°	125° +10°	115° +10°

**Table 2:** The percentage of medial condylar area covered by high stresses (>3,75 MPa) at different neck-shaft and anteversion angles.

Anteversion angle	Neck-shaft angle		
	115°	125°	135°
10°	1%	0%	0%
0°		14%	
−10°	20%	16%	21%
